# Effects of Differential Strategies of Emotion Regulation

**DOI:** 10.3390/brainsci9090225

**Published:** 2019-09-05

**Authors:** Stephanie Boehme, Stefanie C. Biehl, Andreas Mühlberger

**Affiliations:** Department of Psychology, Clinical Psychology and Psychotherapy, University of Regensburg, Universitätsstraße 31, D-93053 Regensburg, Germany

**Keywords:** emotion regulation, electroencephalography, skin conductance response

## Abstract

Patients suffering from mental disorders, especially anxiety disorders, are often impaired by inadequate emotional reactions. Specific aspects are the insufficient perception of their own emotional states and the use of dysfunctional emotion regulation strategies. Both aspects are interdependent. Thus, Cognitive Behavioral Therapy (CBT) comprises the development and training of adequate emotion regulation strategies. Traditionally, reappraisal is the most common strategy, but strategies of acceptance are becoming more important in the course of advancing CBT. Indeed, there is evidence that emotion regulation strategies differ in self-reported effectiveness, psychophysiological reactions, and underlying neural correlates. However, comprehensive comparisons of different emotion regulation strategies are sparse. The present study, therefore, compared the effect of three common emotion regulation strategies (reappraisal, acceptance, and suppression) on self-reported effectiveness, recollection, and psychophysiological as well as electroencephalographic dimensions. Twenty-nine healthy participants were instructed to either reappraise, accept, suppress, or passively observe their upcoming emotional reactions while anxiety- and sadness-inducing pictures were presented. Results showed a compelling effect of reappraisal on emotional experience, skin conductance response, and P300 amplitude. Acceptance was almost as effective as reappraisal, but led to increased emotional experience. Combining all results, suppression was shown to be the least effective but significantly decreased emotional experience when thoughts and feelings had to be suppressed. Moreover, results show that greater propensity for rumination differentially impairs strategies of emotion regulation.

## 1. Introduction

Almost a century ago, Freud determined that there was a downside to emotional inhibition by remarking that the “…inclination to look away and to repress from [...] consciousness the thing that frightens…” considerably contributes to psychological illness (p.246 [1]). The strategy of emotion suppression was repeatedly classified as contributing to mental illness, and it is still blamed for it today (e.g., [2]). At the same time, the successful regulation of emotions may, in fact, be conducive to our well-being. It is also of special interest in social contexts when emotion regulation (ER) can safeguard us against social exclusion and thus enhance one’s survival. People usually possess different strategies of ER and attempts of ER take place automatically and without awareness (e.g., [3]). Commonly, emotions are only perceived as problematic when they are of the wrong intensity, the wrong type, the wrong duration, or when ineffective strategies of ER are applied to them, as it is the case in patients with mental illness [4].

Previous researchers defined several strategies of ER that differ in (mal)adaptiveness [5,6], identifying suppression as a less adaptive strategy of ER. Simultaneously, suppression seems to be the most commonly employed method to deal with undesired emotions (e.g., [6,7]). It was previously shown that successful emotion suppression is associated with decreased behavioral expression, but subjective emotional experience seems to remain unchanged [8,9] or is even increased (e.g., [10]). Sympathetic activation was found to be reduced by suppression in comparison to non-suppression (but see [11]), but seems to be less effective in sympathetic downregulation than other ER strategies (e.g., [12,13]). An appropriate method of measurement of sympathetic activity is electrodermal activity (EDA) or phasic skin conductance response (SCR), respectively [14,15]. Reduced EDA was shown during suppression vs. non-suppression (e.g., [10,16,17]), but the actual subject of suppression seems crucial (e.g., [18]). In laboratory settings, participants were instructed to concentrate on suppressing their overt emotional expression in a way that others would not recognize which emotion they felt (e.g., [8,13]). Research in clinical populations, however, suggests that maladaptive emotional suppression is not just the suppression of expressive behavior, it is also the attempt of suppressing the associated thoughts (for an overview see [19]). It is, therefore, unclear if the reported effects of suppressing just overt emotional expression can indeed be generalized to infer the maladaptiveness of emotion suppression in psychopathology. 

In contrast to emotion suppression, the ER strategy of cognitive reappraisal seems to be more adaptive. It dates back to early stress and coping theories (e.g., [20,21]) and had its peak after cognitive interventions were included in Behavioral Therapy (e.g., [22]). Reappraisal was shown to decrease the experience of negative emotion (e.g., [23,24,25,26,27]) and of the associated sympathetic response (e.g., [24,25,27]). In addition, the recognition performance of emotional stimuli seems to be unaffected by emotional reappraisal (see [9,28] but see [29]), whereas suppression leads to impaired recognition [8,9]. 

More recently, there has been a growing interest in the approach of mindfulness-based emotion regulation, i.e., in the awareness, understanding, and acceptance of emotions (e.g., [30]), with the term “acceptance” describing a non-elaborative, non-judgmental, presence-centered awareness in which thoughts, feelings, and sensations are accepted as they occur [31,32]. Previous research could show that acceptance led to reduced subjective distress (e.g., [13,24]) as well as to a reduction of the associated psychophysiological response [24]. The impact of acceptance on affect is comparable to the impact of reappraisal, but further effects of accepting emotions seem to be less extensive in comparison to strategies of reappraisal or suppression (e.g., [13])

One important aspect of ER research is its effect on neural processes, and one common tool to assess neural activity is to measure electrocortical signal changes. There is ample evidence for electrocortical signal changes after ER. An important event-related brain potential (ERP) is the P300. It was repeatedly shown that P300 amplitudes are associated with subjective stimulus salience and seem to be closely linked to frontal top-down attentional mechanisms as well as to temporo-parietal processes of updating and recognition performance (e.g., [7,33]). Another important ERP in emotional processing is the late positive potential (LPP). Greater LPP amplitudes were associated with more intense/arousing stimuli [34] and decreased LPP amplitudes might reflect neuromodulatory activity, e.g., downstreaming processes in response to increased activation of the amygdala (e.g., [7]). Studies showed decreased LPP amplitudes after suppression (e.g., [35]) and after reappraisal of negative emotional stimuli (e.g., [36,37,38] but see [39,40]). 

To our knowledge, there are only a few studies that compare more than two ER strategies and only a small number of studies that investigate numerous outcomes, such as emotional experience, psychophysiology, electrocortical signal changes, and memory performance. Therefore, the primary aim of this study was to examine the differential effects of three relevant ER strategies (reappraisal, suppression, and acceptance) on subjective experience, psychophysiological (electrodermal), and electrocortical responses as well as on recognition performance. We expected a more pronounced effect of reappraisal on subjective emotional experience than of suppression and acceptance, which should still have a larger effect on subjective emotional experience than passively viewing emotional pictures. We further expected the same pattern for EDA, with the strongest reduction of SCR achieved by reappraisal compared to suppression or acceptance and the lowest reduction achieved by passively viewing emotional stimuli. To our knowledge, there is no study to date examining the effects of ER strategies on P300, although reduced P300 amplitudes are predicted to be caused by the attention-capturing processes, which take place during reappraisal and suppression. We, therefore, hypothesized reduced P300 as well as LPP amplitudes during reappraisal and suppression compared to acceptance and to passively viewing emotional pictures.

Regarding the association between ER and psychopathology, there is evidence that the use of reappraisal strategies is linked to reduced depressive symptoms, whereas the propensity to employ suppression shows opposite effects (e.g., [41,42]). Consequently, the second aim of this study was to assess the impact of psychopathology (increased anxiety and depression scores) on the outcome measures of reappraisal, suppression, and acceptance ER. We expected ER strategies to be less effective in participants who scored higher on questionnaires of depressive and anxious symptomatology.

## 2. Materials and Methods

### 2.1. Participants

Thirty-one participants were recruited via e-mail and public advertisement from the University of Regensburg. One participant canceled participation after recruitment, and due to technical problems during the measurement of electroencephalography (EEG) and psychophysiology, one participant had to be excluded from data analysis. The final sample thus consisted of twenty-nine individuals (25 female). Students of Psychology obtained course credits for their participation. Participants filled in several questionnaires indicating trait and state anxiety (State–Trait Anxiety Inventory: STAI [43]), propensity to rumination (Penn State Worry Questionnaire: PSWQ [44]), and symptoms of depression (Beck Depression Inventory: BDI-II [45]; German version [46]). Table 1 shows the sample description data and the standard/cut-off values of the used questionnaires. STAI and BDI-II standard values are based on the questionnaire manuals, the PSWQ cut-off value for generalized anxiety disorder is based on the work by Salzer et al. [47]. All participants had normal or corrected-to-normal vision and no history of neurological or mental disease. All participants provided written informed consent for the study according to the Helsinki declaration, and the study was approved by the Ethics Committee of the University of Regensburg (18-1074-101).

### 2.2. Stimuli

Stimuli consisted of a subset of anxiety and sadness-inducing pictures from the International Affective Picture System (IAPS [48]). Thirty-six anxiety-inducing (1022, 1040, 1050, 1051, 1052, 1070, 1080, 1090, 1101, 1113, 1120, 1201, 1220, 1230, 1300, 1301, 1302, 1303, 1650, 1930, 1931, 2692, 5972, 6200, 6210, 6230, 6241, 6250, 6260, 6300, 6370, 6510, 6610, 7640, 8480, 9594) and 36 sadness-inducing pictures (2053, 2141, 2205, 2276, 2352, 2580, 2800, 2900, 3080, 3160, 3170, 3220, 3230, 3300, 3301, 3350, 3550, 6570, 9000, 9001, 9005, 9050, 9181, 9220, 9250, 9331, 9400, 9415, 9470, 9561, 9600, 9910, 9911, 9912, 9920, 9921) were chosen based on their ratings of fear and sadness based on the data provided in Libkuman et al. [49]. The mean fear rating for the anxiety-inducing subset was 5.68 (*SD* = 0.78); for the sadness-inducing subset it was 4.15 (*SD* = 0.10), *t* = 7.27; *p <* 0.05. The mean sadness rating for the anxiety-inducing subset was 3.08 (*SD* = 1.20); for sadness-inducing subset it was 6.95 (SD = 0.51), *t* = 17.73; *p <* 0.05. Based on the data by Bradley and Lang [48], the two subsets differed in valence (anxiety-inducing subset: mean rating = 3.67 ± 0.80; sadness-inducing subset: mean rating = 2.64 ± 1.04; *t =* 4.07; *p <* 0.05) and arousal ratings (anxiety-inducing subset: mean rating = 6.13 ± 0.63; sadness-inducing subset: mean rating = 5.32 ± 0.97; *t =* 4.24; *p <* 0.05).

### 2.3. Procedure

During the experiment, participants viewed 16 blocks, each consisting of five anxiety-and five sadness-inducing pictures in randomized order. The ER instruction (“REAPPRAISE”, “SUPPRESS”, “ACCEPT”) and the control instruction “VIEW” were presented before the blocks. Each instruction was thus shown four times, and there were 20 events in each condition (anxiety-inducing and sadness-inducing pictures) per instruction. The meaning of the ER instructions REAPPRAISE, SUPPRESS, ACCEPT, and VIEW was explained to participants both orally and in writing. In the REAPPRAISE condition, participants were requested to cognitively reappraise the meaning of the pictured emotional information in a way that was not linked to their own person. In the SUPPRESS condition, participants were instructed to attempt to suppress all emotional reactions that would normally be produced by the picture information. Similar instructions for SUPPRESS were used in previous studies (e.g., [11,50,51]); however, we additionally asked participants to suppress and downregulate any feelings and thoughts that would be produced by the pictures. In the ACCEPT condition, participants were requested to carefully observe any emotions, thoughts, and expressive behavior produced by the pictures and face them with acceptance, which is in line with mindfulness techniques. In the VIEW condition, participants were requested not to regulate any upcoming feelings, thoughts, and emotional experiences and just view the pictures. Participants were carefully instructed to start regulating their emotional reactions only after picture presentation had started. After briefing and training, the experiment was started when the experimenter had ensured that participants were able to comply with all emotion regulation instructions by asking participants to explain the four instructions in their own words. Otherwise, briefing and training were repeated. 

**Figure 1:**
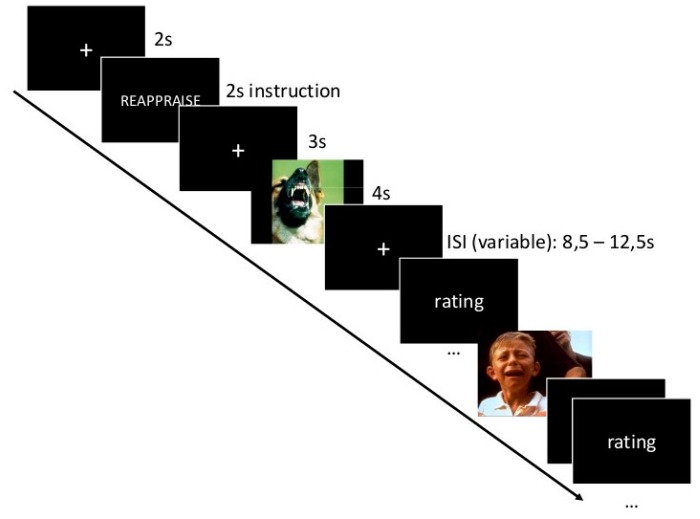
Paradigm: Trial presentation.

The instruction “VIEW”, “REAPPRAISE”, “ACCEPT”, or “SUPPRESS” was presented before each block and 20 pictures (10 anxiety- and 10 sadness-inducing pictures in randomized order) were presented with a jittered inter stimulus interval of 8.5 to 12.5 s. Subsequently, participants rated perceived unpleasantness (1 = very pleasant to 9 = very unpleasant, 5 = neutral) and arousal (1 = not arousing to 9 = very arousing) for each picture on two nine-point Likert scales (Self Assessment Manakin [SAM] [52]). The time course of one trial is depicted in Figure 1. Sixteen anxiety- and sixteen sadness-inducing pictures appeared once in the experiment (resulting in four anxiety- and four sadness-inducing pictures that were shown only once for one of the instructions). Twelve anxiety- and twelve sadness-inducing pictures were shown four times during the course of the experiment (once for each instruction). The four remaining anxiety- and sadness-inducing pictures were not shown during the experiment but appeared during the recognition test after the experiment for the first time. In the recognition test, these “new” pictures were intermixed with the pictures that were presented once in the experimental phase, and participants were requested to indicate if a picture had already been shown before. Responses of the recognition test were transformed into a percentage of right answers across instructions and picture emotions. To avoid effects of stimulus allocation, there were two different versions of the experimental task, with pictures randomly pooled to be presented four times or once or not at all in the ER experiment, with the pictures from the two latter options serving as stimuli during the recognition task. Additionally, the order of instructions differed between the two versions to prevent effects of order.

After the experiment, participants were requested to rate the difficulty they had in their execution of the REAPPRAISAL, SUPPRESSION, ACCEPT, and VIEW instructions on a nine-point-Likert-Scale (1 = very difficult to 9 = very easy). Participants rated VIEW as significantly easier than REAPPRAISAL (*t*_(29)_ = 3.37, *p*_(FDR)_ < 0.01), than ACCEPT (*t*_(29)_ = 2.94, *p*_(FDR)_ < 0.01), and than SUPPRESSION (*t*_(29)_ = 7.27, *p*_(FDR)_ < 0.01). SUPPRESSION was rated as more difficult than REAPPRAISAL (*t*_(29)_ = 2.67, *p*_(FDR)_ < 0.05) and ACCEPT (t_(29)_ = 3.17, *p*_(FDR)_ < 0.01).

### 2.4. Psychophysiological and Electrocortical Recordings and Preprocessing

EEG was measured via standard 32 Ag/AgCl electrodes that were placed on the scalp, based on the 10 to 20 system. AFz served as ground and FCz as reference during recording. Horizontal as well as a vertical electrooculogram (EOG) was recorded by three electrodes. One was placed under the left eye vertically to FP1, creating the bipolar vertical EOG electrode pair. The two remaining electrodes were placed on the outer canthi of both eyes and were linked to form the horizontal EOG. Both horizontal and vertical EOG were subsequently used to remove eye movement artifacts. Movement artifacts in EEG raw data were marked as bad intervals using raw data inspection. Thereafter, data were filtered (Low cutoff: 0.1 Hz, High cutoff: 30 Hz, Notch filter: 50 Hz), corrected for ocular artifacts (following the procedure described by Gratton and Coles [53]), and re-referenced to average recorded reference. Subsequently, data were segmented (200 ms before to 4000 ms after picture onset). To minimize recording and movement artifacts, artifacts were rejected by using common criterions (maximal voltage step = 30 µV/ms, maximal min–max-difference = 100 µV, minimal/maximal amplitude = −1000/1000 µV, interval of low activity = 0.5 µV). Strategies of emotion regulation did not differ in percentage of rejected data (F_(1.08,31.29)_ = 0.54; *p* > .20). Baseline correction was applied by using the 200 ms before stimulus onset as a baseline.

For the P300, several researchers report relative topography changes due to attentional and task demands [54]. Therefore, electrode selection was based on grand average topography and the literature [55] and was restricted to electrodes O1/O2. A visual inspection of the signal time course revealed a time window, which contained all grand average P300 peaks for the different conditions. Therefore, mean amplitudes were extracted for the time window from 240 to 280 ms. To investigate possible LPP differences, averaged mean activations of consecutive time sequences of 200 ms in length were extracted from 300 ms to 1700 ms after stimulus onset (see [37]). Grand average topography showed the LPP to be most pronounced over parietal areas. In accordance with previous studies [7], the signal of P3, P4, Pz, CP1, and CP2 was, therefore, extracted and averaged.

By applying a constant current of 0.5 V, SCR was recorded with two Ag/AgCl electrodes (0.8 cm in diameter and filled with non-hydrating gel) placed on thenar and hypothenar of participants’ nondominant palm. Psychophysiological data were recorded via NeurOne (Mega Electronics Ltd., Kuopia, Finland) and sampled with 1000 Hz. Impedance level was ensured to be less than 10 kΩ. Offline preprocessing was realized with Brain Vision Analyzer 2.1.2 (Brain Products GmbH, Munich, Germany). Raw SCR data was low pass filtered with 1 Hz, segmented, and baseline corrected with the 1000 ms before stimulus onset serving as the baseline. Peak detection was performed 1 to 5 s after stimulus onset. Thereafter, SCR values were z-transformed and subsequently, T-transformed. Due to fast habituation (e.g., [56]), analyses were restricted to the mean of the first four events per condition.

After preprocessing the electrocortical and psychophysiological data, statistical tests were performed with SPSS 25 (IBM Deutschland GmbH, Ehningen, Germany) by conducting repeated measures analysis of variance (ANOVAs). Factors of ANOVAs were the four ER instructions (REAPPRAISE, ACCEPT, SUPPRESS, and VIEW [and additionally NEW for the recognition test]) and the two emotions of the pictures: anxiety and sadness. For LPP F-test, the factor time window was added. In the case of significant F-tests, post-hoc *t*-tests were conducted. To avoid alpha-cumulation, alpha was adjusted using the false discovery rate (FDR [57] and implemented by [58]).

To test for significant influences of enhanced anxiety and proneness to depression (as measure by STAI-trait/PSWQ and BDI-II), bivariate correlation analyses were conducted between the questionnaire scores and the outcomes of subjective emotional evaluation, SCR, P300, and LPP mean amplitude, and recognition performance. Tests were also FDR-corrected [57,58].

## 3. Results

### 3.1. Ratings of Arousal and Unpleasantness

**Figure 2:**
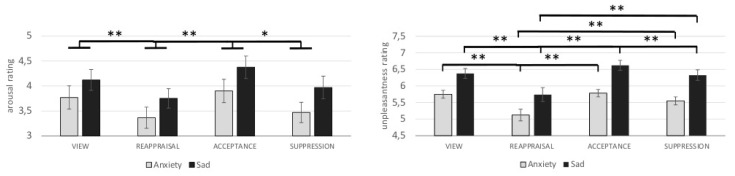
Differential effects (mean and standard errors) of emotion regulation (ER) strategies for anxiety- and sadness-inducing pictures on SAM (Self Assessment Manakin) ratings of arousal (left) and unpleasantness (right). * *p*_(FDR)_ < 0.05; ** *p*_(FDR)_ < 0.01

Each picture was rated for perceived arousal and unpleasantness. The statistical analysis revealed a main effect of ER (arousal: F_(3,87)_ = 8.20, η^2^ = 0.22; unpleasantness: F_(1.72,49.76)_ = 18.40, η^2^ = 0.39) and emotion (arousal: F _(1,29)_ = 19,21, η^2^ = 0.40; unpleasantness: F _(1,29)_ = 48.16, η^2^ = 0.62). The effect ER × Emotion was only significant for ratings of unpleasantness (arousal: F _(1.90,55.13)_ = 0.87 n.s., η^2^ = 0.03; unpleasantness: F _(3,87)_ = 2.72, η^2^ = 0.09). Post-hoc *t*-tests revealed that pictures were rated as less arousing in the REAPPRAISAL than in the VIEW (*t*_(29)_ = 3.27, *p*_(FDR)_ < 0.01) and in the ACCEPT condition (*t*_(29)_ = 4.28, *p*_(FDR)_ < 0.01). Additionally, pictures were rated as less arousing in the SUPPRESSION than in the ACCEPT condition (*t*_(29)_ = 3.08, p_(FDR)_ < 0.01). Comparing arousal ratings between the instructions VIEW and ACCEPT (*t*_(29)_ = 1.28, *p*_(FDR)_ > 0.05), VIEW and SUPPRESSION (*t*_(29)_ = 1.94, p_(FDR)_ > 0.05), and REAPPRAISAL and SUPPRESSION (*t*_(29)_ = 1.87, *p*_(FDR)_ > 0.05) did not reveal significance. Pictures were rated as significantly less unpleasant in the REAPPRAISAL condition than in the VIEW condition (anxiety: *t*_(29)_ = 4.74, p_(FDR)_ < 0.01; sadness: *t*_(29)_ = 4.59, *p*_(FDR)_ < 0.01), in the ACCEPT condition (anxiety: *t*_(29)_ = 4.12, *p*_(FDR)_ < 0.01; sadness: *t*_(29)_ = 5.01, *p*_(FDR)_ < 0.01), and in the SUPPRESSION condition (anxiety: *t*_(29)_ = 3.15, *p*_(FDR)_ < 0.01; sadness: *t_(_*_29)_ = 4.76, *p*_(FDR)_ < 0.01). Additionally, sadness-inducing pictures in the SUPPRESSION condition were rated as less unpleasant than sadness-inducing pictures in the ACCEPT condition (*t*_(29)_ = 3.07, *p*_(FDR)_ < 0.01). Comparing unpleasantness ratings between the instructions VIEW vs. ACCEPT (anxiety: *t*_(29)_ = 0.36, *p*_(FDR)_ > 0.05; sadness: *t*_(29)_ = 2.55, *p*_(FDR)_ > 0.05), VIEW vs. SUPPRESSION (anxiety: *t*_(29)_ = 2.59, *p*_(FDR)_ > 0.05; sadness: *t*_(29)_ = 0.71, *p*_(FDR)_ > 0.05), and ACCEPT vs. SUPPRESSION (anxiety: *t*_(29)_ = 2.51, *p*_(FDR)_ > 0.05) failed significance. Rating data are depicted in Figure 2.

### 3.2. Skin Conductance Response

**Figure 3:**
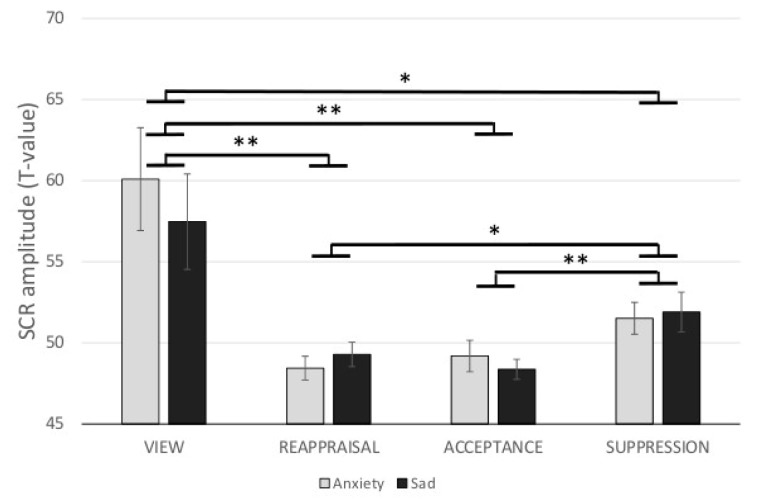
Differential effects (mean and standard errors) of ER strategies for anxiety- and sadness-inducting pictures on skin conductance response (SCR). * *p*_(FDR)_ < 0.05; ** *p*_(FDR)_ < 0.01

For SCR data, there was only a significant main effect of ER (F_(1.26,35.34)_ = 8.91,η^2^ = 0.24). Effects of Emotion (F _(1,28)_ = 1.24 n.s., η^2^ = 0.04) and ER × Emotion (F _(2.21,61.99)_ = 1.41 n.s., η^2^ = 0.05) did not reach significance. Post-hoc *t*-tests showed that SCR-amplitude was reduced in REAPPRAISAL (*t*_(29)_ = 3.22, *p*_(FDR)_ < 0.01), ACCEPT (*t*_(29)_ = 3.25, *p*_(FDR)_ < 0.01), and SUPPRESSION (*t*_(29)_ = 2.49, *p*_(FDR)_ < 0.05) vs. VIEW. Additionally, SUPPRESSION pictures revealed a significant increased SCR amplitude as compared to REAPPRAISAL (*t*_(29)_ = 2.58, *p*_(FDR)_ < 0.05) as well as ACCEPT pictures (*t*_(29)_ = 3.26, *p*_(FDR)_ < 0.01). SCR differed not significantly between the instructions REAPPRAISAL vs. ACCEPT (*t*_(29)_ = 0.10, *p*_(FDR)_ > 0.05). SCR data is depicted in Figure 3.

### 3.3. EEG Data

**Figure 4:**
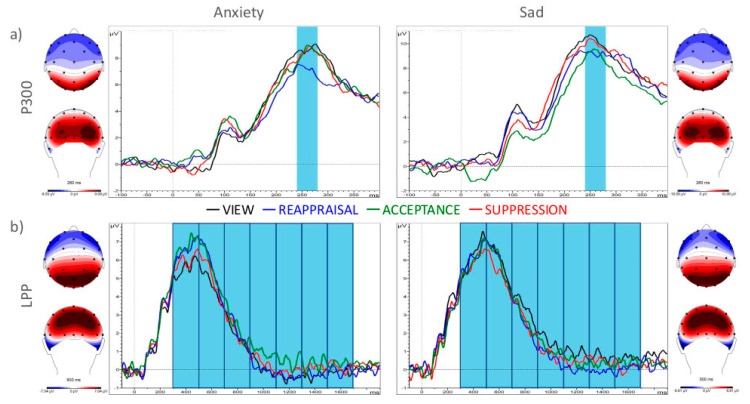
Electroencephalographic results. (**a**) the P300 mean averaged electrocortical signals for anxiety-inducing (left) and sadness-inducing pictures (right); (**b**) the LPP mean averaged electrocortical signals for anxiety-inducing (left) and sadness-inducing pictures (right). Upper column: Differential electrocortical signal (averaged for O1 and O2) of ER strategies for anxiety- (left) and sadness-inducing (right) pictures; lower column: Differential electrocortical signal (averaged for P3, P4, Pz, CP1, and CP2) of ER strategies for anxiety- (left) and sadness-inducing (right) pictures; blue bars depicted extracted time windows.

For P300 amplitudes, analyses of variance revealed significant main effects (ER: F_(3,87)_ = 2.83, η^2^ = 0.09, Emotion: F _(1,29)_ = 5.83, η^2^ = 0.17). The interaction ER × Emotion failed to reach significance (F _(3,87)_ = 0.55, n.s., η^2^ = 0.02). Post-hoc *t*-tests showed significantly reduced P300 mean amplitude for REAPPRAISAL vs. VIEW (*t*_(29)_ = 3.34, *p*_(FDR)_ < 0.05) and vs. SUPPRESSION (*t*_(29)_ = 2.78, *p*_(FDR)_ < 0.05). There was no significant mean amplitude difference between VIEW vs. ACCEPT (*t*_(29)_ = 1.12, *p*_(FDR)_ > 0.05), VIEW vs. SUPPRESSION (*t*_(29)_ = 0.63, *p*_(FDR)_ > 0.05), REAPPRIASAL vs. ACCEPT (*t*_(29)_ = 2.20, *p*_(FDR)_ > 0.05), and ACCEPT vs. SUPPRESSION (*t*_(29)_ = 0.46, *p*_(FDR)_ > 0.05). Figure 4a depicts the P300 mean averaged electrocortical signals for anxiety-inducing (left) and sadness-inducing pictures (right).

For the analysis of differential LPP mean amplitudes, the additional factor Time (300–500, 500–700, 700–900, 900–1100, 1100–1300, 1300–1500, and 1500–1700 ms) was included in the analyses of variance. Due to a significant interaction of ER × Emotion (F_(3,87)_ = 3.51,η^2^ = 0.11), ER × Time (F_(8.14,236.06)_ = 3.08,η^2^ = 0.01), and Emotion × Time (F_(3.65,105.89)_ = 7.71,η^2^ = 0.21), post-hoc analyses were conducted for the different time windows and instructions for anxiety-inducing and sadness-inducing pictures separately. There were a significantly increased mean amplitude for REAPPRAISAL in comparison to VIEW in two time windows (300–500: *t*_(29)_ = 3.46, *p*_(FDR)_ < 0.01; 500–700: *t*_(29)_ = 2.79, *p*_(FDR)_ < 0.05) and for ACCEPT in comparison to VIEW in four time windows (300–500: *t*_(29)_ = 3.34, *p*_(FDR)_ < 0.01; 500–700: *t*_(29)_ = 2.67, *p*_(FDR)_ < 0.05; 900–1100: *t*_(29)_ = 3.55, *p*_(FDR)_ < 0.01; 1100–1300: *t*_(29)_ = 3.33, *p*_(FDR)_ < 0.05) for anxiety-inducing pictures. Additionally, the mean amplitude was induced during ACCEPT in comparison to REAPPRAISAL between 900–1100 ms (*t*_(29)_ = 2.92, *p*_(FDR)_ < 0.05) for anxiety-inducing pictures. During 300–500 ms mean amplitude for ACCEPT vs. SUPPRESSION was increased (*t*_(29)_ = 2.76, *p*_(FDR)_ < 0.05). Remaining post-hoc *t*-tests did not reach significance (see Supplementary Table S1). Figure 4b depicts the LPP mean averaged electrocortical signals for anxiety-inducing (left) and sadness-inducing pictures (right).

### 3.4. Recognition Test

**Figure 5:**
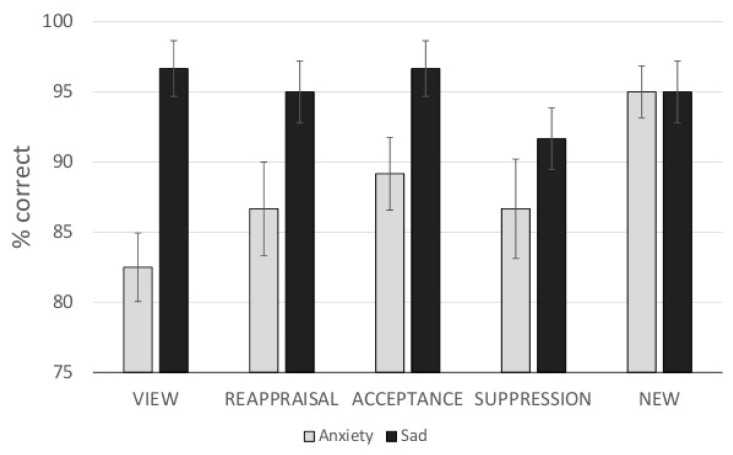
Recognition test: Percentage mean and standard error of correct decisions concerning the familiarity (just seen in the experiment phase) for new pictures and pictures of the view, reappraisal, acceptance, and suppression condition.

Analyzing differential recollection performance, analyses revealed a significant main effect of ER (F_(4,116)_ = 2.54, η^2^ = 0.08) and Emotion (F _(1,29)_ = 18.70, η^2^ = 0.39), indicating that recognition performance was generally increased for sadness-inducing pictures. The interaction of ER × Emotion failed to reach significance (F _(2.87,83.27)_ = 2.36, n.s., η^2^ = 0.08). Post-hoc *t*-tests did not reach significance after alpha-adjustment (NEW vs. SUPPRESSION: *t*_(29)_ = 2.54, *p*_(FDR)_ < 0.10; VIEW vs. NEW: *t*_(29)_ = 2.54, *p*_(FDR)_ < 0.10; VIEW vs. ACCEPT: *t*_(29)_ = 2.11, *p*_(FDR)_ > 0.05; VIEW vs. SUPPRESSION: *t*_(29)_ = 0.162, *p*_(FDR)_ > 0.05; VIEW vs. REAPRRAISAL: *t*_(29)_ = 0.57, *p*_(FDR)_ > 0.05; ACCEPT vs. NEW: *t*_(29)_ = 1.04, *p*_(FDR)_ > 0.05; ACCEPT vs. SUPPRESSION: *t*_(29)_ = 1.51, *p*_(FDR)_ > 0.05; ACCEPT vs. REAPPRAISAL: *t*_(29)_ = 1.22, *p*_(FDR)_ > 0.05; NEW vs. REAPPRAISAL: *t*_(29)_ = 2.07, *p*_(FDR)_ > 0.05, and SUPPRESSION vs. REAPPRAISAL: t_(29)_ = 0.64, *p*_(FDR)_ > 0.05; see also Figure 5).

### 3.5. Effect of Anxiety and Proneness to Depression

To test the effect of enhanced anxiety and proneness to depression (as measure by STAI-trait/PSWQ and BDI-II), correlation analyses were conducted between these three questionnaire scores and rating data, SCR amplitudes, P300, and LPP mean amplitudes and correct responses on the cued recall test. Because of type I error accumulation, only effects with a *p*-value < 0.01 are reported here. Increased PSWQ-scores were correlated with an increased SCR-response to anxiety-inducing pictures in the REAPPRAISAL condition (*r =* 0.51, *p < 0.01*) as well as with increased unpleasantness ratings of anxiety-inducing pictures in the SUPPRESSION condition (*r =* 0.49, *p < 0.01*).

## 4. Discussion

We found evidence for a differential effectiveness of the ER strategies reappraisal, acceptance, and suppression on different outcomes. Results showed significantly reduced subjective arousal to pictures during reappraisal vs. acceptance and view conditions. Similarly, suppression was more effective to reduce arousal ratings than acceptance. For ratings of unpleasantness, *t*-tests showed comparable differences for anxiety- as well as for sadness-inducing pictures. Ratings of unpleasantness showed comparable patterns as ratings of arousal. In addition, these ratings showed significantly reduced scores for reappraisal ER strategy in comparison to acceptance, suppression, and view instruction, thereby underlining the effectiveness of downregulating subjective emotional involvement. This is in accordance with previous findings that showed the superiority of reappraisal [23,24,25,26,27]. In contrast, acceptance does not seem to change the emotional experience compared to the control (view) condition, as arousal and unpleasantness ratings between acceptance and view conditions were not significantly different. This is in line with previous studies, which also reported acceptance to be less effective in reducing emotional experience (e.g., [13,24]). Surprisingly, our suppression instruction was less effective in the downregulation of perceived unpleasantness than reappraisal, but comparably effective in perceived arousal reduction. Compared to previous experiments, which requested participants to suppress solely observable emotional expressive behavior, we used a slightly changed instruction for the suppression condition. Participants in our study were instructed to additionally suppress picture-associated negative thoughts and feelings as this component of ER is better linked to psychopathology (e.g., [59]). Taking the results of Demaree et al. [60] into account, who showed that it is hard to focus only on expressive behavior and that focusing on cognition also reduces the emotional experience, it is possible that the changed instruction of suppression in our study is responsible for the effective reduction in emotional experience during suppression.

Concerning skin conductance, there were reduced mean SCRs for all ER strategies in comparison to the view condition. Additionally, reappraisal and acceptance did not differ in SCR reduction, but both strategies were more effective in the reduction of SCR when compared with suppression. This is in line with other findings, which found sympathetic reduction in reappraisal and acceptance, with suppression being less effective in psychophysiological downregulation (e.g., [8,13]). So far, our results indicate that reappraisal shows a predominant downregulation effect on subjective emotional experience and sympathetic activity. To accept the emotional reactions has no effect on subjective emotional experience reduction, but downregulates the psychophysiological reaction as effectively as reappraisal does. This pattern of unchanged subjective emotional response during the acceptance of negative emotional information might be the reason why patients indicate that acceptance is the most difficult strategy to deal with undesirable emotions [61], although our psychophysiological data show that acceptance is less stressful than suppression. The response pattern of suppression is quite the opposite, with an arousal and unpleasantness rating reduction of comparable effectiveness to the view condition, but a less effective reduction in psychophysiological downregulation when compared to reappraisal and acceptance. It is rather interesting that also during suppression, the subjective and objective stress responses are differentially affected, indicating that people might overestimate the usefulness of suppressing undesirable emotions due to subjective emotional experience reduction, which comes at the cost of greater psychophysiological stress (see also [11]).

The early event-related potential P300 was repeatedly associated with subjective stimulus salience [7] and was shown to be increased after viewing emotional stimuli (e.g., [62,63]). Our results showed reduced P300 amplitudes during reappraisal, indicating that cognitively reappraising emotional pictures changes processing even during relatively early stages of information processing. Although several authors predict reduced P300 amplitudes during reappraisal (e.g., [7]), this is to our knowledge, the first study which showed these differential P300 mean amplitudes. Concerning LPP amplitudes, significant differences between ER strategies were restricted to anxiety-inducing pictures. Here, mean amplitudes for reappraisal and acceptance were increased in the early time windows (300–500 ms and 500–700 ms) and for acceptance vs. view and acceptance vs. reappraisal in later time windows (900–1300 ms). The LPP is a late ERP that is linked to neuromodulatory processes [64] and is larger in response to arousing pictures (e.g., [63,65]). Our results are in line with results from Krendl et al. [39], or Grecucci [40], but contrary to the majority of earlier studies which showed a decreased LPP for reappraisal [36,37,66]. A possible reason for these heterogeneous findings may be differing cognitive demands in reappraising emotional information, which may be reflected in LPP amplitude differences. For instance, Foti and Hajcak [66] showed that prompting reappraisal thoughts and therefore, reducing cognitive demands during reappraisal reduces LPP amplitudes.

Additionally, we were interested in the effects of ER on memory performance and therefore, conducted a recognition test after the experiment. Several authors report unchanged or slightly increased recognition performance for more adaptive strategies, e.g., reappraisal, than for maladaptive ones, e.g., suppression (e.g., [28,67]). The F-test revealed a significant effect of ER instruction, but post-hoc *t*-tests failed to reach significance after FDR-correction. Additionally, there was a significant effect of emotion type, indicating that sadness-inducing pictures were better remembered than anxiety-inducing pictures. This might reflect a deeper processing and easier gate into memory for more negative and arousing stimuli. Consistently, participants rated sadness-inducing pictures as more negative and more arousing than anxiety-inducing pictures.

To summarize the presented effects of the investigated strategies of ER, our results highlight the ER strategy of reappraisal as most effective concerning subjective emotional experience, sympathetic downregulation, and dampening of attentional capture as reflected in altered subsequent information processing. Interestingly, acceptance leads to comparable effects concerning skin conductance response, although emotional experience was increased. Concerning the electrocortical response pattern of acceptance, results for the later ERP (LPP) showed comparably increased LPP mean amplitudes for acceptance than for reappraisal, which even exceeded the latter in later time windows. Taken together, patterns for acceptance and reappraisal are similar, except for emotional experience and P300 mean amplitudes. The suppression instruction in our study (to not only suppress overt emotional expression) led to a reduction of emotional experience that was comparable to that of reappraisal, although sympathetic activation was less decreased compared to reappraisal and acceptance. The electrocortical response did not differ between suppression and the view condition, which indicates that suppressing emotional information seems to be an effective strategy of ER with regard to the emotional experience. However, given the electrocortical similarity to non-emotion regulation (passively viewing), this effect might be of short duration.

Finally, we were interested in possible effects of trait anxiety and symptoms of depression. We found a significantly increased SCR during reappraisal and an increased rating of unpleasantness during suppression in more rumination-prone participants. Both findings were restricted to anxiety-inducing pictures. This may lead to the assumption that in anxiety-prone individuals, the sympathetic downregulation of reappraisal is impaired while the perceived unpleasantness of emotional information during suppression is increased. This might indicate that differential constructs are affected by trait anxiety, which could lead to higher sympathetic activation during more sophisticated emotion regulation strategies (e.g., cognitive reappraisal) and increased perceived unpleasantness during suppression in more anxious individuals. This may then contribute to hyperarousal and a depressive interpretation bias, both of which are important symptom triggers in anxiety and affective disorders [68].

We would also like to mention some limitations of our study. First of all, the sample size is quite small compared to other studies (e.g., [10,11,13]). In addition, the number of trials was relatively low, and therefore, effects for EEG and especially the recognition test may have been underestimated. However, there is evidence that the findings regarding LPP amplitude are a stable outcome (see [69]). Nevertheless, questions of generalizability of our findings from a relatively young and healthy sample remain. Furthermore, using pictures from the IAPS could be criticized. On the one hand, the depicted anxiety-inducing topics are less variable than the sadness-inducing ones. Anxiety-inducing pictures repeatedly depicted snakes, spiders, and dogs, which might be responsible for the impaired memory performance. On the other hand, it would be interesting if the patterns of effectiveness found here could be reproduced by applying ER to other stimulus material (e.g., auditory stimuli) or to threatening situations (e.g., Trier Social Stress Test [70]).

## 5. Conclusions

Our results highlight the predominant effectiveness of reappraisal compared to suppression and acceptance. Acceptance seems to be an effective and useful strategy as well, especially in contexts where cognitive reappraisal is not possible. Our results also indicate that suppression can be effective when it is not restricted to overt emotion expression. Moreover, we could show that enhanced propensity of rumination impairs differential effects of reappraisal and suppression and is could be a crucial factor in the development of mental disorders.

## Figures and Tables

**Table 1:** Characteristics of participants.

	Mean	SD	Standard or Cut-Off Values
age (years)	22.10	5.13	
STAI-state	36.10	4.94	33/34 ^a^
STAI-trait	37.38	7.17	33/34 ^a^
BDI-II	6.10	4.59	≤8
PSWQ	43.07	8.40	≤50

STAI: State-Trait Anxiety Inventory, BDI-II: Beck Depression Inventory, PSWQ: Penn State Worry Questionnaire; ^a^: raw score corresponding to T = 50 for the male/female normative sample.

## Supplementary Materials

The following are available online at https://www.mdpi.com/2076-3425/9/9/225/s1.
