# Supplementary material for: Effects of Differential Strategies of Emotion Regulation
Source: Brain Sci. 2019 Sep 5;9(9):225. doi: 10.3390/brainsci9090225 (PMC6769771; doi:10.3390/brainsci9090225)
Supplement: Supplementary file 1 [file brainsci-09-00225-s001.pdf]

Supplementary Table S1: Post-hoc-t-values for LPP mean amplitude.

Time Window	ER / Emotion	VIEW vs. REAPPRAISAL	VIEW vs. ACCEPT	VIEW vs. SUPPRESSION	REAPPRAISAL vs. ACCEPT	REAPPRAISAL vs. SUPPRESSION	ACCEPT vs. SUPPRESSION
300–500	anxiety	3.45**	3.34**	0.89	0.58	1.75	2.76*
	sad	0.77	1.16	1.82	0.19	0.82	0.76
500–700	anxiety	2.79*	2.67*	0.61	0.28	2.23	2.09
	sad	0.15	0.35	2.14	0.16	1.35	1.49
700–900	anxiety	0.92	1.51	0.36	0.43	0.49	1.16
	sad	2.18	1.51	2.08	0.75	0.53	0.13
900–1100	anxiety	0.58	3.55**	0.77	2.92*	1.18	1.94
	sad	2.13	0.89	1.85	1.40	0.36	0.92
1100–1300	anxiety	0.33	3.33*	1.04	2.45	0.76	1.78
	sad	2.46	1.64	1.53	1.33	1.62	0.21
1300–1500	anxiety	0.07	1.97	1.30	1.75	1.06	0.52
	sad	2.11	1.69	0.81	1.21	2.25	0.74
1500–1700	anxiety	0.21	1.74	0.87	1.02	0.58	0.52
	sad	1.43	0.59	1.69	0.94	0.13	0.82

The t-values of the post-hoc-t-tests were depicted for LPP mean amplitude for anxiety- and sadness-inducing pictures for the used time windows. * p(FDR) < .05; ** p(FDR) < .01.
